# The Degree of Global DNA Hypomethylation in Peripheral Blood Correlates with That in Matched Tumor Tissues in Several Neoplasia

**DOI:** 10.1371/journal.pone.0092599

**Published:** 2014-03-20

**Authors:** Anna-Maria Barciszewska, Stanisław Nowak, Mirosława Z. Naskręt-Barciszewska

**Affiliations:** 1 Department of Neurosurgery and Neurotraumatology, Karol Marcinkowski University of Medical Sciences, Poznan, Poland; 2 Institute of Bioorganic Chemistry of the Polish Academy of Sciences, Poznan, Poland; Ospedale Pediatrico Bambino Gesu', Italy

## Abstract

There are no good blood and serum biomarkers for detection, follow up, or prognosis of brain tumors. However, they are needed for more detailed tumor classification, better prognosis estimation and selection of an efficient therapeutic strategy. The aim of this study was to use the epigenetic changes in DNA of peripheral blood samples as a molecular marker to diagnose brain tumors as well as other diseases. We have applied a very precise thin-layer chromatography (TLC) analysis of the global amount of 5-methylcytosine (m^5^C) in DNA from brain tumors, colon and breast cancer tissues and peripheral blood samples of the same patients. The m^5^C level in tissue DNA from different brain tumor types, expressed as R coefficient, changes within the range of 0.2–1.6 and overlaps with R of that of blood samples. It negatively correlates with the WHO malignancy grade. The global DNA hypomethylation quantitative measure in blood, demonstrates a big potential for development of non-invasive applications for detection of a low and a high grade brain tumors. We have also used this approach to analyze patients with breast and colon cancers. In all these cases the m^5^C amount in DNA cancer tissue match with data of blood. This study is the first to demonstrate the potential role of global m^5^C content in blood DNA for early detection of brain tumors and others diseases. So, genomic DNA hypomethylation is a promising marker for prognosis of various neoplasms as well as other pathologies.

## Introduction

Cancer results from the accumulation of genetic and epigenetic mutations in a susceptible cells [1]. Among neoplasms brain tumors comprise the most malignant group, usually diagnosed at the late stage of the disease, with limited treatment possibilities and poor prognosis. The most abundant group of primary brain tumors are gliomas, followed by meningiomas. A comprehensive appreciation of the integrated genomics and epigenomics of gliomas is urgently needed for better understanding of the multiple cellular pathways involved in brain tumor development, and establishing markers of resistance to traditional therapies as well as contributing to the development of new treatment modalities The neuropathology system classifies brain tumors according to their morphological resemblance to the corresponding glial cells, cytoarchitecture and immunohistological properties [2]. Currently this approach is the method of choice for typing and grading of brain tumors. Histopathological evaluation of tumor specimens gained by microsurgical resection or by stereotactic biopsy is a standard diagnostic procedure for patients with brain tumors. Neuroimaging (e.g. MRI) is a superior instrumental approach for disease staging and follow up [3].

The effective management of any malignant neoplasm, and brain tumor particularly, requires a precise diagnosis at an early stage, which defines the urgent need for specific and sensitive biomarkers. In general, a good biomarker should be a chemical compound (probe) specifically relevant to the disease, that can be applied to monitor a current state of the neoplasm [4]. There is a wealth of data, which show that there is a different amount of a biomolecule (marker) in a cancer cell compared to its normal counterparts and it could be measured in order to find a clear correlation with the tumor state. A selective biomarker should also identify the susceptibility risk and would be helpful in diagnosing of the disease, and finally introducing therapeutic interventions in proper time for effective treatment. Although current brain tumor treatments are primarily based on histopathological diagnosis, it is obvious that neoplasms with similar histological characteristics records can exhibit substantial molecular heterogeneity that leads to different clinical phenotypes [5]. Moreover the tumor tissue for pathologic evaluation is obtained through an invasive procedure. It is useless for screening and follow-up strategy. Therefore there's a need for gaining of the pathological profile from more accessible patient's material, e.g. peripheral blood. To date, there are no good blood and serum biomarkers for detection, follow up, or prognosis evaluation for brain tumors [4]. Therefore new markers are needed for detailed tumor classification, better prognosis estimation and choosing of an efficient therapeutic strategy.

Although some genetic markers are known [5] little is known how epigenetic characteristics vary between different cell types in health and disease or among individuals. It is now clear that epigenetic changes in histone modifications and DNA methylation can alter gene expression, affect their function and contribute to gliomagenesis [6], [7]. It is obvious that they have also some diagnostic potential for the early detection of cancer.

The best characterized epigenetic mark is the methyl group at the fifth position of cytosine (5-methyldeoxycytosine, m^5^dC) in DNA [6], [7]. Aberrant methylation is found at the early stages of carcinogenesis and distinct types of cancer exhibit specific patterns of methylation changes [7], [9]. It is known that malignant progression and shorter survival in brain tumor patients are associated with the global loss of cytosine methylation (hypomethylation) [10], [11]. That can happen through oxidative stress, which is implicated in the etiology of cancers [12]–[15]. The stress results from a cellular imbalance in the production of reactive oxygen species (ROS) and antioxidant enzyme activities. ROS are formed during normal metabolic processes and also after exposure to oxidizing agents present in the living environment. Under physiological conditions the balance exists among ROS production and scavenging, oxidative alteration of cellular components and their repair.

The nucleic acids are among the macromolecules that are covalently modified by ROS. The most reactive of ROS, hydroxyl radical (•OH), causes a wide range of DNA lesions including canonical and odd bases, deletions, strand breakage, and chromosomal rearrangements [6]. These are blamed for the physiological changes associated with cancer. One of the best studied DNA damage product, 8-oxo-7,8-dihydroguanine (8-oxoGua), is a marker of oxidative stress, and is formed in DNA via a direct reaction of guanosine with •OH [16], [17]. Potentially, the same random radical reaction can take place with all normal and modified DNA constituents, including 5-methyldeoxycytosine in eukaryotic DNA [8], [9]. It is assumed that ca. 5% of all cytosine residues or 1% of bases in the mammalian genomes are methylated. Although DNA methylation has been viewed as a stable epigenetic mark, studies in the past decade have revealed that it is not the case. Out of that, 5% of m^5^C deaminates to thymine under moderately acidic conditions, but 2–5% is converted to thymine during the standard overnight incubation with sodium bisulfite [18]. 5-methylcytosine is a target for hydroxyl radical (•OH), the most reactive ROS [18]. The oxidized m^5^C derivatives are unstable, what results in the loss of methyl group of m^5^C and decrease of global (genomic) m^5^C contents in DNA (demethylation, hypomethylation) [19]. Therefore a global genomic methylation level can be used to detect and distinguish cancerous and benign brain tumor tissues, and to find correlations with their pathological features such as stage, grade, and recurrence [20]–[21].

In order to determine a minute content of m^5^C in DNA in limited amount of available brain tumor tissues and in peripheral blood, we have applied the postlabeling method with [γ-^32^P] ATP of enzymatic DNA hydrolysate and identification of labeled m^5^C with two dimensional thin layer chromatography (TLC) [21]. We have observed a significant loss of m^5^C and found a direct correlation of global m^5^C content in DNA of tumor tissues and their malignancies [21]. In this paper we extended these studies and show that the level of DNA hypomethylation in peripheral blood and tumor tissues of brain tumor patients overlaps. This finding prompted us to use the peripheral blood DNA methylation as a probe for monitoring of the process of carcinogenesis. Furthermore with such a marker one can diagnose and predict the occurrence of brain tumor as well as other diseases.

## Materials and Methods

### Blood and Tissue Samples

The brain tumor tissues were sampled from 183 patients that underwent tumor resection at the Department of Neurosurgery and Neurotraumatology of the University of Medical Sciences in Poznań between 2007 and 2012. The breast and colon cancer tissues were sampled after surgical resection at the Department of Surgical Oncology of Wielkopolska Center of Oncology in Poznań [24]. The peripheral blood from patients with arterial hypertension, seniors, as well as from control group (healthy subjects) was taken at the Department of Cardiology of the University of Medical Sciences in Poznań [23].

Tumor tissues and peripheral blood samples were directly frozen and stored at −80°C. Brain tumor tissues were analyzed in the Laboratory of Neuropathology to determine histological types and grades according to the 2007 WHO classification criteria [2]. The breast and colon cancer tissues were evaluated at the Laboratory of Oncological Pathology [24].

Patient's age was documented at the time of the initial diagnosis. Other demographic and survival data were obtained from the patient's medical records.

Blood and tissue analysis was approved by the Bioethical Committee of University of Medical Sciences, Poznań. All participants provided written consent and indicated willingness to donate their blood and tissue samples for research.

### Isolation of DNA from Tumor Tissue

Genomic DNA was extracted from frozen tumor tissue samples with commercial Genomic Mini kit of A&A Biotechnology, Gdańsk, Poland.

Shortly, 15 mg of wet weight of tissue sample was incubated first with proteinase K and then with RNase A. After centrifugation (15000 rpm for 3 min), the supernatant was applied to mini column. DNA bound to the column was eluted with Tris-buffer pH 8.5 and stored at −20°C for further analysis.

### Isolation of DNA from Peripheral Blood

DNA was isolated from 5 ml of human blood taken into EDTA-covered tubes by lysis with 5–10 ml cold (4°C) buffer of 155 mM NH_4_Cl, 10 mM KHCO_3_, and 0.1 mM Na_2_EDTA pH 7.4 in 0.5 h. The cell lysate in 2.5 ml of buffer containing 75 mM NaCl, 1 mM Na_2_EDTA, pH 8.0, was digested with 2.5 μl protease K solution (10 μg/μl) and 25 μl 20% SDS for 16 h at 55°C. Then 2.5 μl of RNase A (10 μg/μl) was added and incubation continued in 55°C for 2h. Finally 150 μl 5 M NaCl was added and tube shaked vigorously for 15 min, then centrifuged at 14000 rpm for 15 min. The DNA was precipitated with two volumes of cold ethanol, removed with pipette and dissolved in 100 μl of water [22]. The purity of DNA preparations was checked by measuring of UV absorbance at 260 and 280 nm. The A_260_/A_280_ ratio was 2.0–2.1.

### DNA Hydrolysis, Labeling and TLC Chromatography

DNA (dried, 1 μg) was dissolved in a succinate buffer (pH 6.0) containing 10 mM CaCl_2_ and digested with 0.001 units of spleen phosphodiesterase II and 0.02 units of micrococcal nuclease in 3.5 μl total volume for 5 h at 37°C. 0.17 μg of DNA digest was labeled with 1 μCi [γ-^32^P]ATP (6000 Ci/mmol; Hartmann Analytic GmbH) and 1.5 units of T4 polynucleotide kinase in 3 μl of 10 mM bicine-NaOH pH 9.7 buffer containing 10 mM MgCl_2_, 10 mM DTT, and 1 mM spermidine. After 0.5 h at 37°C 3 μl of apyrase (10 units/ml) in the same buffer were added and incubated for another 0.5 h. The 3′ nucleotide phosphates were cleaved off with 0.2 μg RNase P1 in 500 mM ammonium acetate buffer, pH 4.5. Identification of [γ-^32^P]m^5^dC was performed by a two-dimensional thin-layer chromatography on cellulose plates (Merck, Darmstadt, Germany) using solvent system: isobutyric acid:NH_4_OH:H_2_O (66∶1∶17 v/v) in the first dimension and 0.1 M sodium phosphate (pH 6.8)-ammonium sulfate-*n*-propyl alcohol (100 ml/60 g/1.5 ml) in the second dimension. Radioactive spot analysis was done with the PhosphoImager Typhoon Screen (Pharmacia, Uppsala, Sweden) and Image Quant Software. The analysis was repeated 3 times and results were evaluated with the statistic software. For precise calculation we have used amount of material in spots corresponding not only to m^5^dC but also to product of its degradation as dC (cytosine) and dT (thymine). Amount of m^5^C was calculated as R = (m^5^dC/m^5^dC+dC+dT)×100 [25], [26].

### Statistical Analysis

STATISTICA software was used for the statistical analyses of all data. Standard deviations were indicated as errors bars on graphs.

## Results

**Figure 1:**
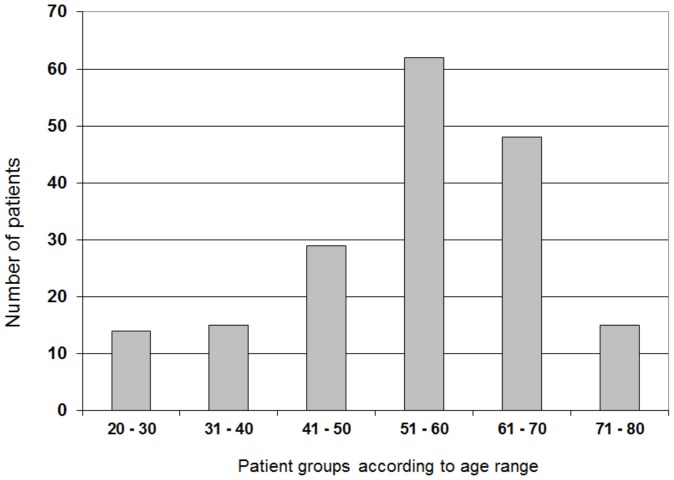
Patients with brain tumors analyzed in this study classified according to age. The 183 patients were divided into 6 groups of different age. The largest group consisted of patients within the age range of 51–60 years.

In this paper we have analyzed 183 individuals with brain tumor aged from 22 to 77. The largest group consisted of 51–70 year-old patients and have the peak incidence in the 6^th^ decade of life (Figure 1). Totally, there were 94 (51.4%) males and 89 (48.6%) females (Table 1). The control group of healthy subjects aged 19–50 years was also included.

**Table 1:** The list of brain tumor types identified in 183 patients for whom DNA from brain tumor tissue and peripheral blood samples was isolated and analyzed for the content of m^5^C in DNA.

Case	Brain tumor histological type	WHO Grade	Sex	Age	R tissue	R blood
1	Fibrillary astrocytoma	II	M	26	1,54	1,63
2	Fibrillary astrocytoma	II	M	32	1,61	1,41
3	Fibrillary astrocytoma	II	M	32	1,53	1,41
4	Fibrillary astrocytoma	II	F	35	1,54	1,49
5	Fibrillary astrocytoma partially gemistocytic	II	F	29	1,35	1,40
6	Fibrillary astrocytoma partially protoplasmatic et gemistocytic	II	F	29	1,33	1,31
7	Fibrillary astrocytoma partially protoplasmatic et gemistocytic	II	F	34	1,47	1,42
8	Fibrillary astrocytoma partially protoplasmatic et gemistocytic	II	F	41	1,47	1,34
9	Fibrillary astrocytoma partially gemistocytic	II	F	42	1,31	1,40
10	Fibrillary astrocytoma recurrent, partially gemstocytic with tending to AA	II/III	F	48	1,43	1,28
11	Fibrillary astrocytoma partially protoplasmatic	II	M	51	1,31	1,26
12	Anaplastic astrocytoma	III	F	24	1,05	1,16
13	Anaplastic astrocytoma	III	M	30	0,99	1,02
14	Anaplastic astrocytoma, partially granulocellular	III	M	30	1,05	0,91
15	Anaplastic astrocytoma	III	F	34	1,04	1,01
16	Anaplastic astrocytoma	III	F	35	1,05	0,98
17	Anaplastic astrocytoma, recurrent	III	M	37	1,00	0,74
18	Anaplastic astrocytoma, recurrent	III	M	39	1,00	0,84
19	Anaplastic astrocytoma	III	F	40	0,99	0,84
20	Anaplastic astrocytoma	III	F	44	1,02	1,10
21	Anaplastic astrocytoma	III	F	46	1,03	1,14
22	Anaplastic astrocytoma	III	F	48	1,04	1,03
23	Anaplastic astrocytoma	III	F	49	1,01	1,04
24	Anaplastic astrocytoma	III	M	51	1,07	1,00
25	Anaplastic astrocytoma	III	M	53	1,03	0,73
26	Anaplastic astrocytoma	III	F	54	1,06	1,01
27	Anaplastic astrocytoma	III	F	56	1,04	0,97
28	Anaplastic astrocytoma	III	M	59	1,01	0,94
29	Anaplastic astrocytoma	III	M	68	0,99	1,29
30	Anaplastic astrocytoma	III	M	73	1,05	1,13
31	Anaplastic astrocytoma, recurrent, with tendency to GBM	III/IV	F	56	0,81	0,90
32	Anaplastic astrocytoma	III	M	62	1,02	0,92
33	Glioblastoma	IV	F	22	0,58	0,43
34	Glioblastoma	IV	F	28	0,38	0,91
35	Glioblastoma	IV	F	29	0,22	0,51
36	Glioblastoma, recurrent	IV	M	30	0,49	0,45
37	Glioblastoma	IV	F	35	0,53	0,56
38	Glioblastoma, recurrent	IV	F	39	0,48	0,89
39	Glioblastoma	IV	M	45	0,41	0,47
40	Glioblastoma	IV	M	46	0,43	1,11
41	Glioblastoma	IV	F	47	0,49	0,62
42	Glioblastoma	IV	F	48	0,56	0,56
43	Glioblastoma	IV	M	48	0,52	0,12
44	Glioblastoma	IV	M	49	0,38	0,40
45	Glioblastoma	IV	M	49	0,68	0,88
46	Glioblastoma	IV	F	51	0,52	0,29
47	Glioblastoma, recurrent	IV	F	52	0,52	0,92
48	Glioblastoma, recurrent	IV	M	53	0,62	0,88
49	Glioblastoma	IV	M	53	0,34	0,39
50	Glioblastoma	IV	M	54	0,46	0,76
51	Glioblastoma	IV	M	54	0,46	0,50
52	Glioblastoma, recurrent	IV	M	54	0,53	0,74
53	Glioblastoma, recurrent	IV	F	54	0,32	0,56
54	Glioblastoma	IV	F	54	0,42	0,33
55	Glioblastoma	IV	M	54	0,43	0,51
56	Glioblastoma	IV	F	55	0,34	0,50
57	Glioblastoma	IV	M	56	0,46	0,55
58	Glioblastoma	IV	F	57	0,37	0,51
59	Glioblastoma	IV	F	57	0,47	0,45
60	Glioblastoma	IV	M	58	0,50	0,34
61	Glioblastoma	IV	M	59	0,40	0,55
62	Glioblastoma	IV	M	60	0,34	0,71
63	Glioblastoma	IV	F	62	0,51	0,81
64	Glioblastoma	IV	M	62	0,51	0,82
65	Glioblastoma	IV	F	64	0,53	0,37
66	Glioblastoma	IV	F	65	0,51	0,64
67	Glioblastoma, recurrent	IV	F	65	0,42	0,37
68	Glioblastoma	IV	M	66	0,25	0,27
69	Glioblastoma	IV	M	66	0,55	0,49
70	Glioblastoma	IV	M	66	0,38	0,53
71	Glioblastoma	IV	M	67	0,53	1,12
72	Glioblastoma, recurrent	IV	M	67	0,59	0,68
73	Glioblastoma	IV	F	67	0,29	0,68
74	Glioblastoma recurrent	IV	M	68	0,53	0,66
75	Glioblastoma	IV	M	69	0,46	0,56
76	Glioblastoma	IV	F	69	0,55	0,41
77	Glioblastoma	IV	F	70	0,16	0,35
78	Glioblastoma	IV	F	71	0,39	0,70
79	Glioblastoma	IV	M	71	0,35	0,57
80	Glioblastoma	IV	M	71	0,50	0,32
81	Glioblastoma recurrent	IV	F	74	0,48	0,44
82	Glioblastoma	IV	M	75	0,56	0,53
83	Glioblastoma	IV	M	75	0,31	0,61
84	Recurrent anaplastic gliomas partially granulocellular	III	M	45	0,49	0,60
85	Anaplastic glioma	III	M	60	0,70	0,55
86	Giant cell glioblastoma recurrent	IV	F	45	0,44	1,13
87	Giant cell glioblastoma	IV	M	46	0,41	0,88
88	Oligodendroglioma	III	F	53	1,14	0,90
89	Isomorphia oligodendroglioma	II	F	58	1,18	0,97
90	Isomorphia oligodendroglioma	II	M	67	1,22	1,37
91	Anaplastic oligodendroglioma, recurrent	III	M	31	0,98	1,21
92	Anaplastic oligodendroglioma, recurrent	III	M	57	1,08	0,82
93	Anaplastic oligodendroglioma	III	F	64	0,98	0,81
94	Anaplastic oligoastrocytoma (mixed glioma)	III	F	22	1,24	0,92
95	Anaplastic oligoastrocytoma	III	M	47	0,97	0,75
96	Anaplastic oligoastrocytoma (mixed glioma)	III	M	48	1,13	0,80
97	Anaplastic ependymoma	III	M	40	1,30	1,14
98	Central neurocytoma	II	M	25	1,17	1,26
99	Neurinoma I	I	F	29	1,26	1,35
100	Neurinoma I (schwannoma)	I	F	31	1,31	1,28
101	Meningothelial meningioma	I	F	28	1,56	1,55
102	Meningothelial meningioma	I	F	47	1,62	1,69
103	Meningothelial meningioma	I	F	48	1,64	1,46
104	Meningothelial meningioma	I	F	51	1,59	1,75
105	Meningothelial meningioma	I	F	52	1,55	1,65
106	Meningothelial meningioma	I	F	52	1,57	1,52
107	Meningothelial meningioma	II	F	57	1,13	1,48
108	Meningothelial meningioma	I	F	58	1,61	1,51
109	Meningothelial meningioma	I	F	63	1,58	1,63
110	Meningothelial meningioma	I	F	65	1,52	1,63
111	Meningothelial meningioma	I	M	67	1,56	1,68
112	Angiomatous meningioma	I	M	51	1,43	1,33
113	Angiomatous meningioma	I	F	60	1,48	1,51
114	Angiomatous meningioma	I	M	61	1,49	1,45
115	Angiomatous meningioma	I	F	66	1,51	1,64
116	Angiomatous meningioma	I	F	67	1,54	1,59
117	Fibrous meningioma	I	F	55	1,54	1,51
118	Fibrous meningioma partially psammomatous	I	F	61	1,41	1,57
119	Fibrous meningioma	I	M	63	1,71	1,67
120	Fibrous meningioma	I	F	66	1,57	1,68
121	Fibrous meningioma	I	M	71	1,62	1,67
122	Atypical meningioma	II	F	54	1,46	1,45
123	Atypical meningioma	II	M	65	1,53	1,44
124	Atypical meningioma	II	M	77	1,43	1,35
125	Anaplastic meningioma	III	F	43	1,42	1,26
126	Anaplastic meningioma	III	F	73	0,99	0,95
127	Meningothelial meningioma transitionale	I/II	F	67	1,54	1,67
128	Transitional meningioma partially psammomatous	I	F	69	1,53	1,63
129	Transitional meningioma	I	F	71	1,48	1,56
130	Haemangiopericytoma	III	M	45	1,13	0,98
131	Haemangioblastoma	I	F	34	1,18	1,26
132	Metastatic tumor (ovary)	G2	F	52	1,23	0,64
133	Metastatic tumor (ovary)	-	F	61	0,99	0,71
134	Metastatic tumor (primary site not defined)	-	F	64	0,47	0,43
135	Metastatic tumor (colon)	G3	M	51	0,31	0,24
136	Metastatic tumor (rectum)	-	M	60	0,50	0,69
137	Metastatic tumor (brest)	G2	F	51	0,70	0,98
138	Metastatic tumor (brest)	-	F	58	0,97	1,19
139	Metastatic tumor (kidney)	-	M	56	0,49	1,24
140	Metastatic tumor (lung)	-	F	48	0,59	0,58
141	Metastatic tumor (lung)	-	F	49	0,32	0,49
142	Metastatic tumor (lung)	G2	M	49	0,86	0,70
143	Metastatic tumor (lung)	-	M	49	0,58	0,97
144	Metastatic tumor (lung)	-	M	50	0,82	0,88
145	Metastatic tumor (lung)	-	M	51	1,01	1,18
146	Metastatic tumor (lung)	G3	M	51	0,75	0,54
147	Metastatic tumor (lung)	G3	M	52	0,85	0,76
148	Metastatic tumor (lung)	G3	F	54	0,12	0,36
149	Metastatic tumor (lung)	G3	F	54	1,29	0,38
150	Metastatic tumor (lung)	-	F	54	1,09	0,92
151	Metastatic tumor (lung)	G3	M	54	0,72	0,94
152	Metastatic tumor (lung)	G3	M	55	0,38	0,36
153	Metastatic tumor (lung)	G2	F	56	0,85	0,67
154	Metastatic tumor (lung)	G2	M	57	0,64	0,65
155	Metastatic tumor (lung)	-	M	58	0,46	0,52
156	Metastatic tumor (lung)	G2	M	58	0,87	0,86
157	Metastatic tumor (lung)	G3	M	59	0,61	0,78
158	Metastatic tumor (lung)	-	M	60	0,98	0,96
159	Metastatic tumor (lung)	-	M	60	0,59	0,43
160	Metastatic tumor (lung)	-	M	61	0,52	0,43
161	Metastatic tumor (lung)	G2	F	61	1,03	0,82
162	Metastatic tumor (lung)	-	M	61	0,82	0,61
163	Metastatic tumor (lung)	G3	M	62	0,60	0,36
164	Metastatic tumor (lung)	G3	F	62	0,27	0,33
165	Metastatic tumor (lung)	G2	M	64	0,84	0,95
166	Metastatic tumor (lung)	-	M	64	0,62	0,68
167	Metastatic tumor (lung)	-	M	64	0,49	1,12
168	Metastatic tumor (lung)	-	F	66	0,26	1,03
169	Metastatic tumor (lung)	G3	M	66	0,66	0,53
170	Metastatic tumor (lung)	G2	F	68	0,77	0,68
171	Metastatic tumor (lung)	G1	M	69	0,93	0,96
172	Metastatic tumor (lung)	G3	M	71	0,46	0,46
173	Metastatic tumor (lung)	-	M	72	0,56	0,33
174	Metastatic tumor (lung)	-	M	73	0,69	0,67
175	Metastatic tumor (melanoma)	IV	F	55	0,57	0,72
176	Metastatic tumor (thyroid)	-	F	54	0,35	0,49
177	Metastatic tumor (primary site not defined)	-	F	52	0,53	1,31
178	Metastatic tumor (primary site not defined)	-	M	62	0,29	1,16
179	Metastatic tumor (primary site not defined)	-	M	65	0,49	0,56
180	Metastatic tumor (primary site not defined)	-	M	69	0,28	0,48
181	Metastatic tumor (primary site not defined)	-	M	77	0,73	0,98
182	Malignant neoplasma	IV	M	49	0,45	0,62
183	Malignant neoplasma	IV	M	53	0,41	0,54

Specific R coefficient was calculated as (m5dC/m5dC+dC+dT)×100 on the basis of analysis TLC plate exposed to Phosphoimager. Histopathological analysis revealed the WHO grade. Sex is also mentioned.

**Figure 2:**
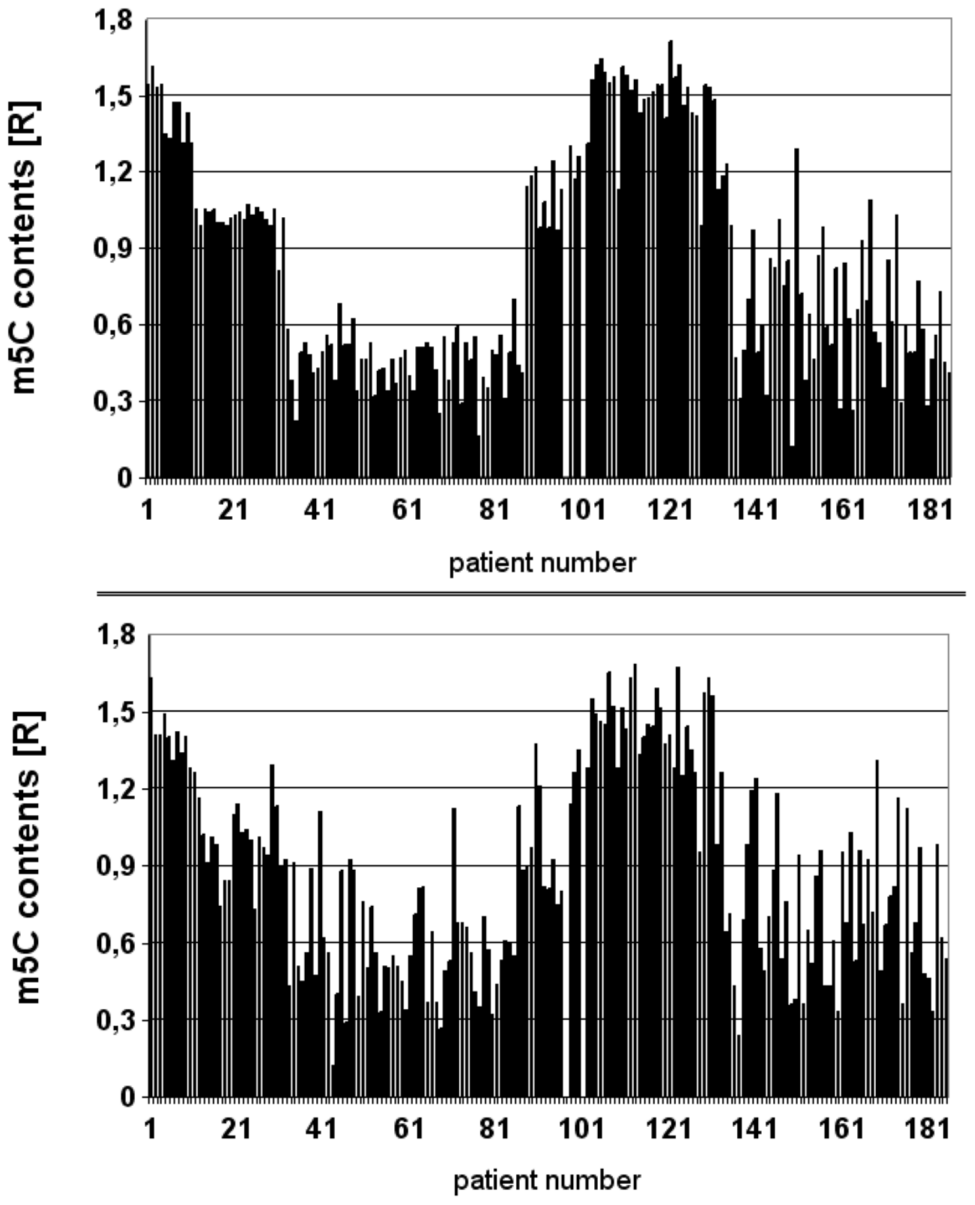
Comparison of content of m^5^C in DNA from tissue and blood. 5-methylcytosine content (R) in DNA from 183 patients with brain tumors: cancer tissues (upper panel) and blood (bottoms panel). The figure clearly shows that m^5^C content, expressed as R [ = (m5dC/m5dC+dC+dT)×100] coefficient, in DNA from tumor tissue matches the results from blood [25], [26].

We analyzed the genomic level of m^5^C in DNA extracted from both brain tumor tissue and peripheral blood sample from all patients (Figure 2). The histopathological evaluation of brain cancer tissues was carried out according to WHO classification and combined with the results of m^5^C content analysis in DNA of tissues and blood (Table 1). As one can see the pattern of m^5^C amount in DNA of brain tumor tissue of 183 patients is almost identical to that of blood of the same individuals (Figure 2, Table 1).

**Figure 3:**
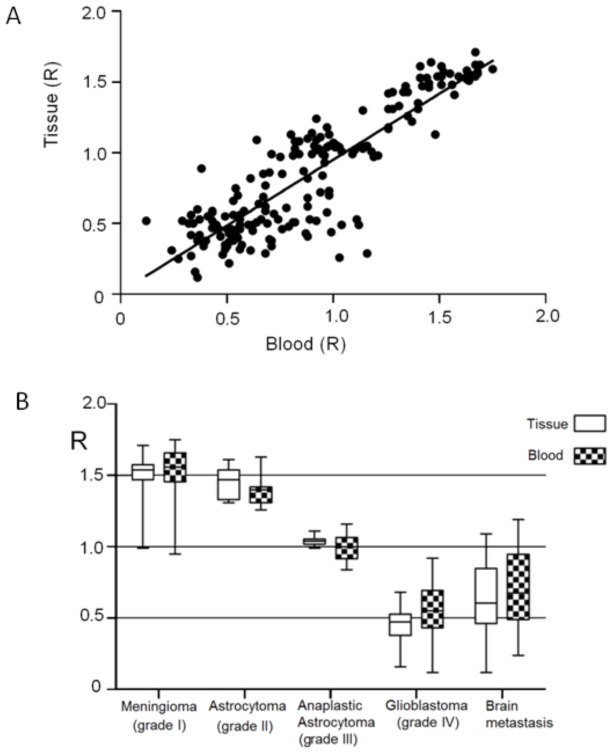
Comparison of m^5^C content in DNA in tissue and blood with malignancy of brain tumors. **A**. Pearson r correlation (0.9; p<0.0001) of genomic m^5^C contents of DNA from peripheral blood and from brain tumor tissues of the same subjects. **B**. Amounts of m^5^C in DNA (R) from blood and in tumor tissues of the same subjects with brain tumors (astrocytoma, anaplastic astrocytoma and glioblastoma) of different malignancy. Data on human brain metastasis and meningioma were also analyzed. Data were evaluated with ANOVA test.

It turned out that m^5^C content in matching tumor tissues and blood samples correlates well with the pathological data and correlates well with the tumor grading. The level of m^5^C in DNA from both samples of the same patient is very similar, if not identical (Figure 2). That result is very striking and never observed before for brain tumors and also for other diseases. In comparison to the cancer tissue, peripheral blood is a much desirable source of biomarkers due to its accessibility. Our results clearly show that the correlation between R values for tissues and blood is very high. This is perfectly confirmed by the Pearson correlation coefficient of 0.9 (Figure 3A). This finding immediately suggests that m^5^C contents in DNA of peripheral blood can be used directly as a diagnostic tool in neurooncology. The other most interesting result of our studies is that the level of m^5^C in DNA of brain tumor tissue and blood of the same brain tumor patient negatively correlates with the tumor malignancy grade (Figure 3B). This means that increasing demethylation is accompanied with the increasing malignancy grade. Fibrillary astrocytoma (WHO grade II) patients showed the R value**s** of m^5^C in DNA around 1.5, but for anaplastic astrocytoma (WHO grade III) the R is ca. 1 for both tumor tissues and blood samples. One can see that the most devastating brain tumor, glioblastoma multiforme (WHO grade IV), is characterized with the R below 0.5. These values are the lowest one for DNA methylation observed ever for a high grade gliomas as well as for other tumors as well. For comparison, we also look at metastatic brain tumors. They show a slightly higher R values, between 0.5–1 (Figure 3B). It means also that m^5^C content in DNA can be good measure for separation of metastatic from other brain tumor Our data for 183 subjects clearly show a linear correlation of m^5^C content in both brain tumor tissue and blood DNA with the WHO malignancy grade (Figure 3B). These results are in good agreement with the previous observations on a smaller group of patients, which showed also reverse correlation of DNA demethylation with tumor grades and their malignancies [21]. Our data are also supported by others that primary glioblastoma and established gliomas' cell lines show significant reduction of m^5^C content in comparison with normal brain tissue [8].

**Figure 4:**
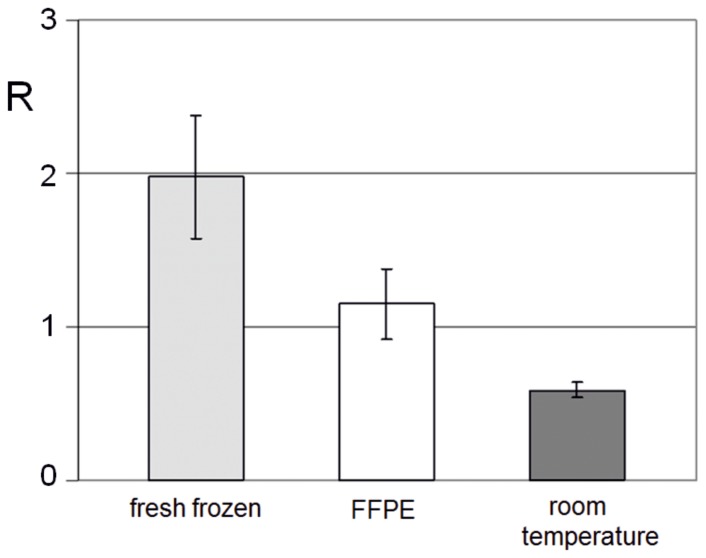
Effect of brain tumor tissue handling on content of m^5^C in DNA. The level of m^5^C content (R) in DNA isolated from resected meningioma tissue (WHO grade I) stored at −80°C (grey bar), formalin-fixed paraffin-embedded (FFPE) (empty bar) and exposed to room temperature for 3 h (black bar). Analysis was done for 5 samples in each conditions. Standard deviations for R is shown.

One should remember that an important issue for all markers is their stability and sensitivity. On the other hand any fresh biological material is not stable and prone to degradation and oxidation. To evaluate that issue, the freshly resected tumor (Meningothelial meningioma) tissue was divided and exposed to three different conditions. One part was fresh-frozen and kept on dry ice after resection, the second was formalin-fixed paraffin*-*embedded (FFPE), and the third part was stored for 3 hours at room temperature. DNA isolated from the differently treated samples showed the highest R value for the deeply fresh-frozen tissues. Significantly higher DNA demethylation (lower R value) of FFPE specimen was observed. For the sample stored at room temperature severe hypomethylation of DNA was found (Figure 4).

It is obvious that only direct deep freezing totally and effectively terminates oxidative (damage) processes after tumor resection. On the other hand paraffin embedding significantly stimulates DNA oxidation damage and demethylation. This finding has to be taken into account during an analysis of FFPE samples.

## Discussion

Several methods can be used to detect the genomic (global) m^5^C content. DNA can be digested into nucleotides and the total m^5^C amount can be quantified by high performance liquid chromatography, or liquid chromatography/mass spectrometry. These methods have some limitations. They need a relatively high amount of tissue and DNA sample (e. g. HPLC chromatography), very expensive, not routine equipment for mass spectrometry and finally specifically labelled m^5^dC. In the case of brain tumors, availability of a tissue is very limited. Both techniques identify of m^5^C in one dimension (a chromatogram). One should add that these approaches are recognized as very laborious techniques and are difficult to be applied directly for the clinical diagnostics [27].

Our approach is based on the post-labeling with [γ-^32^P]ATP and T4 kinase and requires only a minute amount of DNA as in case of brain tumor tissue. In our approach we have measured m^5^C contents in total enzymatic digest of DNA after postlabeling with radioactive ATP and chromatographic separation of nucleotides. This method is quantitative and requires only small amounts of DNA. It is a simple biochemical method, cheap and reliable, easy to be standardized. No specific equipment is needed. The advantage is that it provides global content of m^5^C in a genome, which is based on hydroxyl radical catalyzed DNA demethylation.

Brain tumors comprise a heterogeneous collection of neoplasms, which originate either primarily in the central nervous system or represent metastases from other, extracranial, tumors. The global hypomethylation has been observed in primary glioblastomas and the level of m^5^C varies between GBM ranging from near normal levels to approximately 50% of normal, reflecting the loss of methylation approximately 10^6^ CpG sites per tumor cell [6], [9].

It is known that DNA methylation changes may lead to the genetic instability which is characteristic for cancer. Hypomethylation can be due to hydrolytic deamination and demethylation of m^5^C. Because of critical relations between genomic hypomethylation and pathogenesis, there is a growing interest to determine whether changes in global DNA methylation can be used as a specific biomarker of the disease. Since hematogenous dissemination of the tumor cells is the main mechanism for remote metastasis, peripheral blood DNA analysis may be a feasible approach for detecting systemic tumor cell spreading. Tumor related free methylated DNA in blood of cancer patients has been assessed for its clinical utility. Peripheral blood is a readily available source of genomic DNA that can be used to assess DNA methylation level. There are several reports on blood based methylation biomarkers for various solid tumor types including breast, ovarian, pancreatic, bladder, colorectal and lung cancers [28].

**Figure 5:**
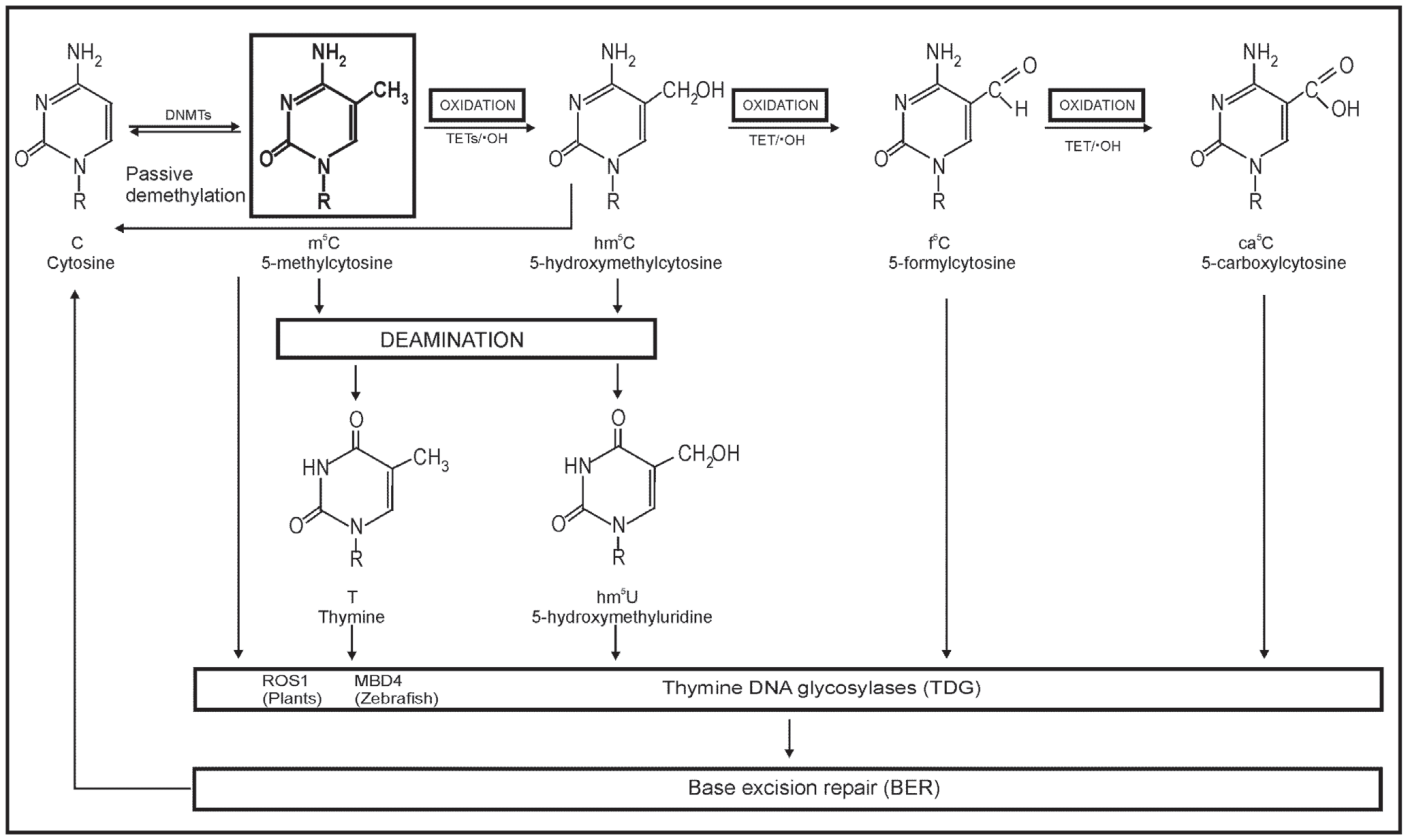
General pathways of 5-methylcytosine decay. The methylated cytosine can be degraded enzymatically or randomly hydroxyl radical (•OH). Two reactions are predominant: a) stepwise oxidation of methyl group with formation of 5-hydroxymethylcytosine, 5-formylcytosine and 5-carboxylcytosine, b) deamination to thymine and to 5-hydroxymethyluridine. All these modified nucleosides are formed on DNA level and can be repaired [13], [14].

It is a very well known and accepted view that the neoplastic transformation is associated with the increased production of reactive oxygen species. The average adult human brain uses a large percentage of the body's total oxygen consumption and generates a large amount of ROS. They can potentially oxidize all components of the cell. DNA components are natural targets for ROS. Guanine is easily oxidized to 8-oxoguanine, which is frequently used as a marker of the genome damage, mostly due to its easy detection with the electrochemical approach [12], [17]. On the other hand, the enzymatic and radical ROS oxidation of, m^5^C in DNA, leads to the formation of 5-hydroxymethylcytosine, 5-formylcytosine and 5-carboxycytosine, which are repaired and finally demethylation of DNA is observed (Figure 5). There are not simple methods for their detection. In addition to these damage products the oxidative deamination of m^5^C also takes place [18]. Therefore these two ROS induced reaction pathways as oxidation and demethylation, cause dramatic structural changes in DNA and have big functional consequences (Figure 5). For example aberrant global DNA methylation can be a consequence of changes in DNA methyltransferases' activity [29].

In this paper we put forward idea that free radicals damage of m^5^C is real and cannot be excluded. It is evidenced by different treatment of a tissue (Figure 4). This approach is new and has not been considered up to now.

Our results are the first to demonstrate that levels of m^5^C in DNA in both brain tumor tissues and matched serum samples are associated with the malignancy grades. To get a detailed insight in total m^5^C content into genomic DNA of tumor and blood, the most abundant brain tumor types were analyzed individually (Figure 2, Table 1). Close inspection of each of them shows a very similar level of m^5^C for grade I meningioma and fibrillary astrocytoma (WHO grade II). A lower amount of m^5^C is due to anaplastic astrocytoma (WHO grade III). The content of m^5^C in DNA of patients with glioblastoma (WHO grade IV) and metastatic brain tumors are much lower. The literature data suggest different origins of the samples or the existence of the subgroups for glioblastoma [30]–[32]. In-deep inspection of our results for glioblastoma clearly shows three levels of m^5^C: very small (R∼0.25), medium (R∼0.5) and high (R∼0.7) (Table 1). Therefore these different R values can correlate with 3 subsets of gliomas: proliferative, mesenchymal and proneural [32]. We analyzed also whether R for DNA from peripheral blood can be used as a diagnostic tool also for other cancers and diseases.

**Figure 6:**
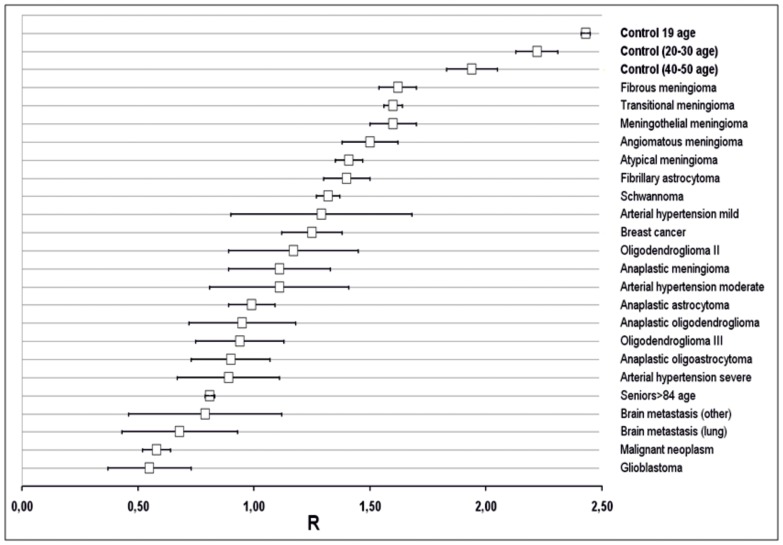
The diagram showing the relation of m^5^C content (R values with deviations errors) in DNA isolated from peripheral blood of patients with different brain tumors, breast and colon cancers and arterial hypertension. As one can see there is strict relation of R with different diseases. R decreases as malignancy increases. The amount of m^5^C suggests a possibility of a disease occurrence. As a control samples from healthy patients of age group 19–50 were used.

Recently, we showed a strong negative correlation of m^5^C contents in DNA and the disease stage of patients with the arterial hypertension [23]. We have got similar results for breast and colon cancer patients [24]. For these diseases we have observed that level of m^5^C in DNA is different and smaller than for brain tumors (Figure 6). We have also measured m^5^C level in healthy individuals of different age (Figure 6). They have showed R value above 2. In general, we observe DNA hypomethylation in neoplasms and other diseases, and the range of these changes is specific for each pathology as one can see in Figure 6.

Because our assay may become a promising screening tool for brain tumors and other diseases, we have to mention its specificity. In the paper we demonstrated that the degree of DNA hypomethylation is characteristic for tumor tissue and blood samples across the brain tumors' malignancy grades and all stages of other neoplasms (Figures 4 and 6). Therefore the collected data confirm usefulness of m^5^C in DNA of the peripheral blood as a cancer biomarker for early detection and diagnosis not only for brain tumors, but also for other human disorders [23], [24], [33].

The degree of the DNA demethylation is specific for particular brain tumor. It is also specific for other non-cancer diseases.

## Conclusion

In summary this paper shows that hypomethylation in DNA of tumor tissue and peripheral blood samples from patients with primary and metastatic brain tumors are almost at the same level. Our results provide compelling evidence for the potential usefulness of DNA methylation as a non-invasive diagnostic method for early detection and prognosis of various diseases.
